# Respiratory health and disease in a UK population-based cohort of 85 year olds: The Newcastle 85+ Study

**DOI:** 10.1136/thoraxjnl-2015-207249

**Published:** 2016-01-05

**Authors:** Andrew J Fisher, Mohammad E Yadegarfar, Joanna Collerton, Therese Small, Thomas B L Kirkwood, Karen Davies, Carol Jagger, Paul A Corris

**Affiliations:** 1 Department of Respiratory Medicine, Newcastle Upon Tyne Hospitals NHS Foundation Trust, Newcastle Upon Tyne, UK; 2 Institute for Ageing, Newcastle University, Newcastle Upon Tyne, UK; 3 Faculty of Medical Sciences, Institute of Health and Society, Newcastle Upon Tyne, UK

**Keywords:** Lung Physiology, COPD epidemiology, Respiratory Measurement

## Abstract

**Background:**

People aged 85 years and older are the fastest growing age group worldwide. This study assessed respiratory health, prevalence of respiratory disease and use of spirometry in respiratory diagnosis in a population-based cohort of 85 year olds to better understand respiratory health and disease in this sector of society.

**Methods:**

A single year birth-cohort of 85 year olds participated in a respiratory assessment at their home or residential institution including self-reporting of symptoms and measurement of spirometry. General practice medical records were reviewed for respiratory diagnoses and treatments.

**Findings:**

In the 845 participants, a substantial burden of respiratory disease was seen with a prevalence of COPD in medical records of 16.6% (n=140). A large proportion of the cohort had environmental exposures through past or current smoking (64.2%, n=539) and occupational risk factors (33.6%, n=269). Spirometry meeting reliability criteria was performed in 87% (n=737) of participants. In the subgroup with a diagnosis of COPD (n=123), only 75.6% (n=93) satisfied Global Initiative in Obstructive Lung Disease (GOLD) criteria for airflow obstruction, and in a healthy subgroup without respiratory symptoms or diagnoses (n=151), 44.4% (n=67) reached GOLD criteria for airflow obstruction and 43.3% (n=29) National Institute of Health and Care Excellence criteria for at least moderate COPD.

**Interpretation:**

Spirometry can be successfully performed in the very old, aged 85 years, and may help identify respiratory diseases such as COPD. However interpretation in this age group using current definitions of COPD based on spirometry indices may be difficult and lead to overdiagnosis in a healthy group with transient symptoms.

**Key messages**

**What is the key question?**

- What is the burden of respiratory disease and utility of spirometry in aiding assessment of respiratory health and diagnosis of respiratory disease in community-living 85 year olds in the UK?

**What is the bottom line?**

- The study reveals a substantial burden of respiratory disease and symptoms in 85 year olds but also considerable discordance between physician-diagnosed COPD and confirmatory spirometry evidence in the very old that have important implications for clinical practice.

**Why read on?**

- This study represents the largest and most detailed assessment to date of respiratory health status and challenges of using spirometry criteria in respiratory diagnosis in the very old, aged 85 years and over, which are now the fastest growing sector of the population.

## Introduction

The very old, aged 85 years and older, are now the most rapidly expanding age sector of most populations worldwide.^1^ Data from the 2011 England and Wales Census showed a doubling of the over 85 years age group between 1985 and 2010, from nearly 0.7 million to over 1.4 million,^2^ and numbers are projected to double again between 2010 and 2030.^3^ This age group frequently uses healthcare resource in primary and secondary care,^4^ and therefore understanding their health status and burden of disease is important for training of health professionals and for organisation of healthcare provision.

Symptoms relating to the respiratory system, in particular dyspnoea, are common in those 85 years and older with a prevalence of over 40%,^5^ and are frequently a reason for older people to seek healthcare. Although it is recognised that many chronic respiratory diseases increase in prevalence and severity with age, it is also clear that dyspnoea is non-specific and may be associated with non-pulmonary morbidities.^6^ In the very old, assessment of respiratory health is further complicated by the physiological changes that occur as part of ‘normal’ or ‘healthy’ ageing, such as loss of lung elasticity and reduced thoracic cage movement, which will have an effect on objective measures of lung function.^7^

Current national and international guidelines on the management of COPD have obstructive spirometry (FEV_1_/FVC ratio <0.7) as a key diagnostic test directing physicians towards the use of specific respiratory medications.^8^ ^9^ However, the accuracy of lung function criteria for the diagnosis of airflow obstruction or restrictive lung disease in very old people has been questioned due to the intrapulmonary and extrapulmonary physiological changes that occur in this age group as part of normal ageing.^10^ This may cause misdiagnosis and inappropriate use of medications in this population. Moreover, a previous study in a population with a mean age of 73 years suggested that COPD may be either overdiagnosed or underdiagnosed depending on the approach taken to defining abnormal lung function.^11^

This study aimed to address the lack of knowledge about respiratory health, prevalence of lung disease and objective measures of lung function in the very old using baseline data from the Newcastle 85+ Study,^4^ ^12^ a large population-based cohort of 85 year olds. Specifically the study aimed to: assess the extent of common respiratory symptoms and the prevalence of physician-diagnosed lung disease, particularly COPD; and to assess the accuracy of COPD diagnosis based on lung function measurements, respiratory symptoms and identification of risk factors, and the degree to which respiratory medication was appropriately prescribed. Finally, in a healthy reference group (HRG), the study aimed to evaluate the applicability of three standard methods of interpreting lung function measurements as normal or abnormal to disentangle the effects of lung disease and ‘normal’ or ‘healthy’ ageing on measured lung function.

## Methods

Full details of the Newcastle 85+ Study methodology have been reported.^12^ In brief, members of the 1921 birth cohort living in Newcastle upon Tyne or North Tyneside (North-East England) were recruited around their 85th birthday over a 17 month-period spanning 2006 and 2007. Participants included people living at home or in institutional care and regardless of their current health status. More detailed methods are available as online supplementary materials.

### Existing diagnoses of respiratory disease, respiratory symptoms, respiratory medications and environmental risk factors

Current and past respiratory diagnoses were identified from a general practice records review (GPRR) using a predetermined checklist of chronic respiratory diseases. Data on use, but not doses, of respiratory medications were also obtained from GPRR. Data on symptoms of breathlessness, cough, wheeze and sputum production were obtained by a structured questionnaire administered as part of a domiciliary multidimensional health assessment (MDHA) conducted by a research nurse in the participant's home or institution. Specifically, participants were asked whether shortness of breath limited their day-to-day activities and responses were then used to assign an Medical Research Council (MRC) dyspnoea score.^8^ Participants were asked about any relevant environmental exposure in their occupation or at home, specifically detailed smoking history and relevant occupational history (including exposure to heavy industry generally as well as the chemical industry, asbestos and coal mining). Two measures of disease burden were used: a disease count (maximum 18 diseases) previously determined in the cohort; and a non-respiratory disease count excluding COPD and other respiratory disease (maximum 16 diseases).^4^ Further details of the individual respiratory diagnoses, medications and chronic non-respiratory diseases included in the disease count are provided (see online supplementary methods).

### Lung function measurements

Spirometry and peak flow measurements were performed at the participant's place of residence by a trained research nurse using MicroLab Spirometer and Spida V.5 software (Micro Medical, Rochester, UK). The aim was to obtain three technically satisfactory maximal effort ‘blows’ to generate reproducible FEV_1_, FVC and peak expiratory flow measurement (PEF); blows were repeated until this was achieved or maximum effort reached. Blows were assessed for technical adequacy using in-built Spida algorithms. All spirometry curves were assessed independently by a respiratory clinical physiologist and those able to produce at least two adequate blows were included in the analysis. If the necessary quality was lacking they were excluded from analysis. Demispan was measured as a surrogate for height^13^ (calculated using standard equations) and height used with age and gender to calculate predicted values for FEV_1_, FVC and peak flow using equations in the UK Department of Health guide.^9^ Spirometry was classified (see online supplementary table S2) as normal, obstructive or restrictive based on the FEV_1_/FVC ratio of 0.7 and the percentage of predicted values for FEV_1_ and FVC, with obstructive spirometry further classified as mild, moderate or severe based on Global Initiative in Obstructive Lung Disease (GOLD) criteria.^10^ In addition, we reanalysed the data using criteria presented by the Global Lung Function Initiative (GLI)^14^ which provides alternative prediction model equations validated for ages 3 years to 95 years (see online supplementary tables S3–S5).

### Healthy reference group

To establish the distribution of normal lung function in people aged 85 years, we identified a HRG of participants with no respiratory symptoms, no respiratory diagnoses, no current use of respiratory medications and no non-respiratory diagnosis which might influence lung function (eg, Parkinson's disease, kyphoscoliosis, heart failure, ankylosing spondylitis) in their GPRR. Those with a BMI >30 were also excluded from HRG. Lung function in the HRG was compared against equation derived^15^ predicted values based on gender and height by three accepted methods: percentage predicted value; lower limit of normal (LLN) using American Thoracic Society/European Respiratory Society (ATS/ERS) criteria;^16^ and Z scores.

### Statistical methods

Gender differences in respiratory symptoms, diagnoses, environmental exposures and medications were examined using χ^2^ and Mann-Whitney U tests. Gender differences in lung function were investigated in the whole sample, COPD group and the HRG using Mann-Whitney U test for continuous measures, χ^2^ and Fisher's exact tests for categorised measures and Kruskal-Wallis test for ordered categorised measures. The relationship between FEV_1_ and PEF scores was assessed using Pearson's correlation coefficients. Sensitivity analyses were carried out to examine differences between those included and excluded from analysis due to lack of spirometry measures and those with and without an MRC dyspnoea score. All analyses were conducted using Stata V.12.0 (StataCorp; College Station, Texas, USA).

## Results

### Sociodemographic, non-respiratory health characteristics and environmental exposures of the study population

**Figure 1:**
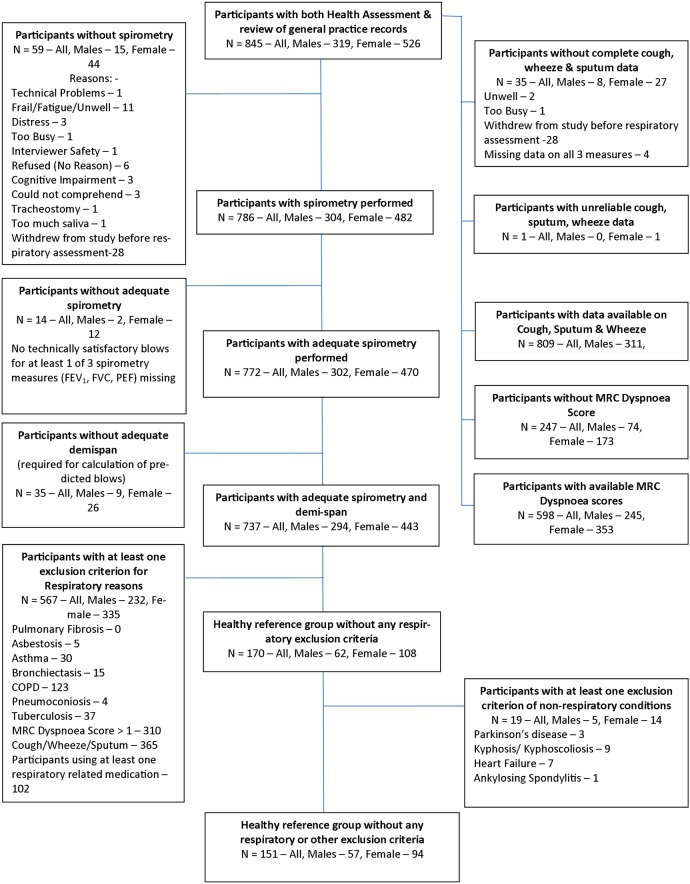
Flow chart illustrating how the total cohort of Newcastle 85+ Study participants was subdivided in the respiratory study sample, demonstrating why different numbers of participants are included in the analyses. The derivation of the study groups are shown in the flow chart; note that for some variables the number of participants included is less than 845 due to missing data, the reasons for which are detailed. The basis for the healthy reference group (HRG) was the 845 participants who had multidimensional health assessment (MDHA) and general practice records review (GPRR) conducted. Of these, 786 (93.0%) had spirometry performed of whom 772 performed it adequately; a further 35 participants with missing demispan were removed (unable to calculate predicted blows), resulting in 737. Participants with at least one respiratory condition, those with respiratory symptoms and those on respiratory medication were excluded which reduced the group size to 170. Other conditions which have an effect on spirometry values were also taken into account leading to exclusion of a further 19 participants. The remaining 151 (17.9% of 845) participants formed the HRG.

Details of the Newcastle 85+ Study population have been reported previously, and the study population was broadly sociodemographically representative of the local population, and of England and Wales, including the proportion in institutional care.^4^ Data from MDHA and GPRR was available for 845 participants, 58.2% (845/1453) of those eligible (figure 1); their mean (SD) age was 85.5 (0.4) years, 62.3% (526/845) were female and 99.6% (839/845) were of white ethnic group (table 1). Three-quarters were living in standard housing, 12.8% (108/845) in warden-supported accommodation and 10.2% (86/845) in institutional care. The median (IQR) chronic disease count was 5(3–6) with no significant gender difference (p=0.074). Although the 845 participants were a non-random sample of the eligible population, data from an additional 188 participants (18%) who opted for GPRR only showed no difference in respiratory diagnoses compared with those who participated fully.

**Table 1:** Sociodemographic and health characteristics of the total Newcastle 85+ cohort (n=845) and by gender

	Men (n=319)	Women (n=526)	Overall cohort (n=845)	p Value*
Ethnicity % (N)				
White	99.4 (316)	99.8 (523)	99.6 (839)	0.272†
Living arrangements % (N)				
Standard housing	83.4 (266)	73.2 (385)	77.0 (651)	0.002†
Sheltered housing	10.3 (33)	14.3 (75)	12.8 (108)	
Institutional care	6.3 (20)	12.6 (66)	10.2 (86)	
Smoking % (N)				
Never	25.6 (81)	42.0 (220)	35.8 (301)	<0.001†
Former	69.9 (221)	51.5 (270)	58.5 (491)	
Current	4.4 (14)	6.5 (34)	5.7 (48)	
Occupational exposures % (N)				
Heavy industry	41.2 (126)	16.6 (83)	25.9 (209)	<0.001†
Coal mining	11.4 (35)	0.0 (0)	4.3 (35)	<0.001‡
Chemical industry	11.1 (34)	4.0 (20)	6.7 (54)	<0.001†
Asbestos exposure	28.9 (88)	1.6 (8)	12.0 (96)	<0.001†
Respiratory symptoms % (N)				
Cough	28.3 (88)	25.8 (129)	26.7 (217)	0.425†
Wheeze	25.0 (78)	20.2 (101)	22.0 (179)	0.109†
Sputum production	40.7 (127)	28.0 (140)	32.9 (267)	<0.001
MRC dyspnoea score % (N)				
1	50.2 (123)	40.5 (143)	44.5 (266)	0.048§
2	11.4 (28)	19.0 (67)	15.9 (95)	
3	20.4 (50)	17.6 (62)	18.7 (112)	
4	15.1 (37)	17.0 (60)	16.2 (97)	
5	2.9 (7)	6.0 (21)	4.7 (28)	
Respiratory diagnoses % (N)				
COPD	17.9 (57)	15.8 (83)	16.6 (140)	0.429†
Asthma	6.9 (22)	12.7 (67)	10.5 (89)	0.007†
Bronchiectasis	2.5 (8)	1.5 (8)	1.9 (16)	0.308†
Pulmonary fibrosis	0.0 (0)	0.2 (1)	0.1 (1)	1.000‡
Asbestosis	1.6 (5)	0.0 (0)	0.6 (5)	0.008‡
Pneumoconiosis	1.3 (4)	0.0 (0)	0.5 (4)	0.020‡
TB	4.4 (14)	4.9 (26)	4.7 (40)	0.713†
Respiratory medications				
Inhaled short-acting β-2 adrenoreceptor agonists	9.1 (29)	11.4 (60)	10.5 (89)	0.288†
Inhaled muscarinic antagonists	3.8 (12)	3.8 (20)	3.8 (32)	0.976†
Oral theophylline	0.3 (1)	0.5 (3)	0.5 (4)	0.598‡
Combination short-acting bronchodilators	0.6 (2)	0.0 (0)	0.2 (2)	0.142‡
Inhaled corticosteroids	5.3 (17)	7.8 (41)	6.9 (58)	0.169†
Combination inhaled Corticosteroids and long-acting β-2 adrenoreceptor agonists	1.9 (6)	2.1 (11)	2.0 (17)	0.833†
Oral leukotriene receptor antagonists	0.0 (0)	0.4 (2)	0.2 (2)	0.529‡
Oral mucolytics	0.6 (2)	0.2 (1)	0.4 (3)	0.560‡
At least one respiratory medication				
% (N)	12.2 (39)	14.5 (76)	13.6 (115)	0.361†
Disease count				
median (IQR)	4 (3–6)	5 (4–6)	5 (3–6)	0.074§
Comorbid disease count				
median (IQR)	4 (3–6)	5 (4–6)	5 (3–6)	0.047§

*Comparison of men and women.

§Mann–Whitney U test.

†χ^2^ test.

‡Fisher's exact test,.

Denominators vary due to missing values.

Almost three quarters (74.4%, 235/316) of men and over half of women (58.0%, 304/524) had smoked in their lifetime, although very few (men: 4.4%, 14/316; women: 6.5%, 34/524) were current smokers. A significant proportion of men and women had occupational exposures which may have influenced respiratory health, with much higher prevalence in men (heavy industry: 41.2%, 126/306; coal mining: 11.4%, 35/307; chemical industry: 11.1%, 34/306; asbestos: 28.9%, 88/305), reflecting common historical occupations in this region of the UK (table 1).

### Respiratory diagnoses, symptomatology and medication use

The most common physician-diagnosed respiratory condition was COPD with a prevalence of 16.6% (140/845) and no significant gender difference (p=0.43) (table 1). A diagnosis of asthma had been made in 10.5% (89/845) with a predominance in women (men: 6.9%; women: 12.7%; p=0.007). Other respiratory diagnoses were rare.

Chronic cough was self-reported in 26.7% (217/812) and wheeze in 22.0% (179/812) of participants. Regular sputum production was more common in men (men: 40.7%, 127/312; women: 28.0%, 140/500; p<0.001). An MRC dyspnoea score was assigned in 598 (70.8%) participants since in the other participants their activity could be limited by other non-respiratory conditions. Half (123/245) of the men and 40.5% (143/353) of the women allocated an MRC dyspnoea score had no limitations to their daily activities due to breathlessness.

The most frequently prescribed respiratory medications were inhaled short-acting β-2 adrenoreceptor agonists (10.5%, 89/845 of participants) followed by inhaled corticosteroids (6.9%, 58/845) (table 1). Only 2.0% (17/845) were taking a combination inhaler containing corticosteroid and a long-acting β-2 adrenoreceptor agonist. The use of other respiratory medications was unusual (table 1).

### Lung function measurements

Spirometry was performed by 786 (93.0%) participants (figure 1), most of whom (98.2%, 772/786) provided at least two adequate blows conforming to ATS/ERS guidelines. Demispan was available for 737 participants with adequate expiratory effort and consistency allowing calculation of predicted spirometry values, with these 737 forming the spirometry group (table 2). Comparison of the spirometry group (n=737) with those excluded due to missing/inadequate spirometry and/or missing demispan (n=108) showed those excluded were more likely to be female, living in an institution and with previous exposure to the chemical industry, but not significantly different in smoking history; respiratory symptoms, diagnoses or medications; or dyspnoea scores (see online supplementary table S1).

**Table 2:** Results of spirometry in the cohort completing spirometry with adequate reproducible blows and demispan available for calculation of predicted blows (n=737)

	Men (n=293)	Women (n=444)	All (n=737)	p Value*
Actual spirometry median (IQR)				
FEV_1_ (l/s)	1.8 (1.4–2.2)	1.2 (1.0–1.5)	1.4 (1.1–1.8)	<0.001†
FVC (l/s)	2.7 (2.2–3.2)	1.8 (1.4–2.1)	2.0 (1.6–2.6)	<0.001†
FEV_1_/FVC	0.7 (0.6–0.8)	0.7 (0.6–0.8)	0.7 (0.6–0.8)	0.006†
PEF (L/m)	441 (323–604)	283 (196–362)	328 (233–450)	<0.001†
% predicted median (IQR)				
FEV_1_	78.8 (62.4–94.3)	83.4 (68.1–98.8)	81.5 (65.6–97.1)	0.008†
FVC	83.4 (70.3–99.6)	96.6 (79.1–113.7)	90.8 (74.1–108.4)	<0.001†
Spirometry % (N)				
Normal	28.0 (82)	33.3 (148)	31.2 (230)	0.108‡
Restrictive	13.7 (40)	16.2 (72)	15.2 (112)	
Obstructive	58.4 (171)	50.5 (224)	53.6 (395)	
Grading of obstructive spirometry§ % (N)				
Mild	35.7 (61)	43.3 (97)	40.0 (158)	0.059¶
Moderate	46.8 (80)	45.1 (101)	45.8 (181)	
Severe	14.6 (25)	9.8 (22)	11.9 (47)	
Very severe	2.9 (5)	1.8 (4)	2.3 (9)	
FEV_1_ % (N)				
Below LLN	25.9 (76)	13.3 (59)	18.3 (135)	<0.001**
Normal range	73.7 (216)	85.6 (380)	80.9 (596)	
Above ULN	0.3 (1)	1.1 (5)	0.8 (6)	
FEV_1_ Z-score				
median (IQR)	1.0 (0.2–1.7)	0.6 (0.0–1.2)	0.8 (0.1–1.4)	<0.001†
FVC % (N)				
Below LLN	21.2 (62)	9.2 (41)	14.0 (103)	<0.001‡
Normal range	77.1 (226)	86.3 (383)	82.6 (609)	
Above ULN	1.7 (5)	4.5 (20)	3.4 (25)	
FVC Z-score				
median (IQR)	0.9 (0.0–1.5)	0.1 (−0.6–0.9)	0.4 (−0.4–1.2)	<0.001†
Oxygen saturation				
median (IQR)	97 (96–98)	97 (96–98)	97 (96–98)	0.513†

*Comparison of men and women.

†Mann–Whitney U test.

‡χ^2^ test.

§This is based on the 395 participant subsample with obstructive spirometry.

¶Kruskal–Wallis test.

**Fisher's exact test.

LLN, lower limit of normal; PEF, peak expiratory flow; ULN, upper limit of normal.

**Figure 2:**
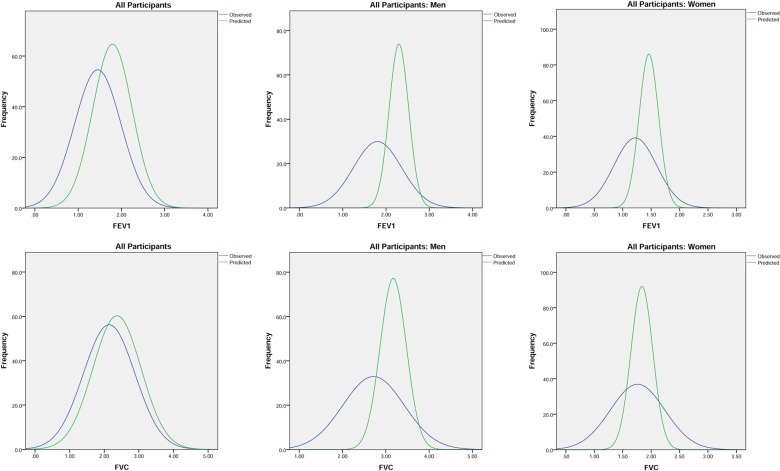
Distribution curves of FEV_1_ and FVC in all participants in spirometry cohort (all, men and women) measured (blue) and predicted (green).

**Figure 3:**
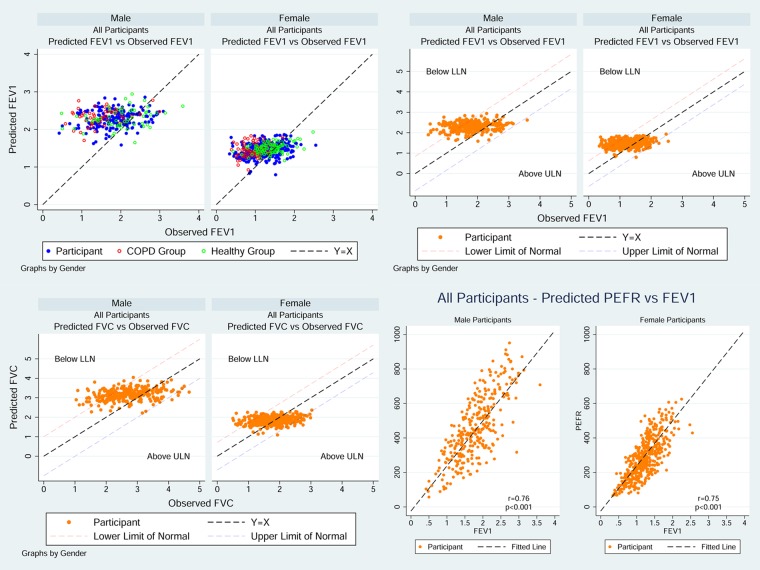
Scatter plots of spirometry and peak expiratory flow in all participants in spirometry cohort.

Of the whole spirometry group, 31.2% (230/737) had a normal FEV_1_/FVC ratio and 15.2% (112/737) had a restrictive pattern. Obstructive spirometry was the most common finding (men: 58.4%, 171/293; women: 50.5%, 224/444) but with no gender difference in the spread of severity (table 2). Measured values of FEV_1_, FVC and PEF in the spirometry group were normally distributed but with a much wider distribution range than that of the predicted values (figure 2). Scatter plots of the measured FEV_1_ and FVC against the predicted values showed more participants with measured values below the predicted values than above suggesting a downward shift in the population as a whole (figure 3). The spread of FEV_1_ measurements around the predicted values was much wider in men than women.

### Prevalence and accuracy of physician-diagnosed COPD

Of the spirometry group, 16.7% (123/737) had physician-diagnosed COPD (COPD group) of whom 57.7% (71/123) were female and 23.8% (29/123) reported being ‘never smokers’ (table 3). More than half of the ‘never smokers’ with a COPD diagnosis had no occupational exposures either.

**Table 3:** Descriptive characteristics of subset with physician-diagnosed COPD in general practitioner records

	Men (n=52)	Women (n=71)	All (n=123)	p Value*
Smoking % (N)				
Never	21.2 (11)	25.7 (18)	23.8 (29)	0.637†
Former	67.3 (35)	67.1 (47)	67.2 (82)	
Current	11.5 (6)	7.1 (5)	9.0 (11)	
Occupational exposure % (N)				
Heavy industry	49.0 (25)	19.7 (14)	32.0 (39)	0.001†
Coal mining	17.7 (9)	0.0 (0)	7.4 (9)	<0.001‡
Chemical	13.7 (7)	2.8 (2)	7.4 (9)	0.034‡
Asbestos	33.3 (17)	7.1 (5)	18.2 (22)	<0.001†
Non-smokers with no occupational exposures % (N)	3.9 (2)	18.3 (13)	12.2 (15)	0.023†
Respiratory symptoms % (N)				
Cough	46.2 (24)	53.5 (38)	50.4 (62)	0.419†
Wheeze	53.9 (28)	56.3 (40)	55.3 (68)	0.784†
Sputum production	63.5 (33)	54.3 (38)	58.2 (71)	0.310†
MRC dyspnoea score % (N)				
1	26.8 (11)	12.5 (7)	18.6 (18)	0.035§
2	9.8 (4)	16.1 (9)	13.4 (13)	
3	34.2 (14)	19.6 (11)	25.8 (25)	
4	22.0 (9)	33.9 (19)	28.9 (28)	
5	7.3 (3)	17.9 (10)	13.4 (13)	
Comorbid respiratory diagnoses % (N)				
Asthma	25.0 (13)	49.3 (35)	39.0 (48)	0.006†
Bronchiectasis	7.7 (4)	2.8 (2)	4.9 (6)	0.240‡
Asbestosis	7.7 (4)	0.0 (0)	3.3 (4)	0.030‡
Pulmonary fibrosis	0.0 (0)	0.0 (0)	0.0 (0)	–
Pneumoconiosis	3.9 (2)	0.0 (0)	1.6 (2)	0.177‡
TB	5.8 (3)	9.9 (7)	8.1 (10)	0.516‡
Medications % (N)				
Inhaled short-acting β-2 adrenoreceptor agonists	36.5 (19)	52.1 (37)	45.5 (56)	0.087†
Inhaled muscarinic antagonists	17.3 (9)	22.5 (16)	20.3 (25)	0.477†
Oral theophylline	1.9 (1)	4.2 (3)	3.3 (4)	0.637‡
Combination short-acting bronchodilators	1.9 (1)	0.0 (0)	0.8 (1)	0.423‡
Inhaled corticosteroids	17.3 (9)	38.0 (27)	29.3 (36)	0.013†
Combination inhaled corticosteroids and long-acting β-2 adrenoreceptor agonists	11.5 (6)	12.7 (9)	12.2 (15)	0.849†
Oral leukotriene receptor antagonists	0.0 (0)	1.4 (1)	0.8 (1)	1.000‡
Oral mucolytics	1.9 (1)	1.4 (1)	1.6 (2)	1.000‡
Oral glucocorticoid therapy	5.8 (3)	4.2 (3)	4.9 (6)	0.697‡
At least 1 respiratory medication % (N)	46.2 (24)	66.2 (47)	57.7 (71)	0.026†
Disease count				
median (IQR)	5 (4–7)	6 (5–7)	6 (4–7)	0.156§
Non-respiratory disease count				
median (IQR)	5 (4–6)	6 (5–7)	6 (4–7)	0.064§

*Comparison of men and women.

†χ^2^ test.

‡Fisher's exact test.

§Mann–Whitney U test.

Denominators vary due to missing values.

In the COPD group, only 45.5% (56/123) were taking short-acting inhaled β-2 adrenoreceptor agonist bronchodilator therapy, 20.3% (25/123) were taking inhaled long-acting muscarinic antagonists, 41.5% (51/123) were on inhaled corticosteroids either as monotherapy (36/51) or in combination with a long-acting β-agonist (15/51). There was minimal use of theophylline preparations, oral mucolytics or oral leukotriene receptor antagonists and none of the COPD group used home oxygen (table 3). The proportion of the COPD group that were on at least one respiratory medication differed significantly between men and women (men: 46.2%, 24/52; women: 66.2%, 47/71; p=0.026), although a sizeable proportion (42.3%, 52/123) of those with a COPD diagnosis were not on any (table 3). There was a significant overlap in the diagnoses of asthma and COPD with 61% (48/78) of those with an asthma diagnosis also being diagnosed with COPD.

Respiratory symptoms were common but not universal in the COPD group with 50.4% (62/123) reporting cough and 58.2% (71/123) sputum production. Nevertheless 26.8% (11/52) of men and 12.5% (7/71) of women with a COPD diagnosis had only minimal breathlessness (MRC dyspnoea score=1).

Only 75.6% (93/123) of the COPD group had obstructive spirometry by GOLD criteria (table 4). There was no gender difference in severity of airflow obstruction (based on % predicted FEV_1_) and only 63.4% (78/123) of the COPD group fulfilled the UK National Institute of Health and Care Excellence (NICE) guidelines spirometry definition of moderate, severe or very severe disease (table 4). Furthermore, only 63.4% (78/123) of the COPD group fulfilled the UK NICE guidelines spirometry definition of moderate, severe or very severe disease. When FEV_1_ was classified by the LLN approach, 48.1% (25/52) of men and 33.8% (24/71) of women from the COPD group fell below the LLN with all other participants falling between the LLN and upper limit of normal, suggesting that a substantial proportion (60.2%, 74/123) of those with physician-diagnosed COPD had an FEV_1_ in the normal range and/or no airflow obstruction on spirometry measurement. When applying the GLI prediction models to the COPD group, 48.1% (25/52) men and 50.7% (36/71) women satisfied criteria for airflow obstruction (see online supplementary table S4). The degree of agreement between physician-diagnosed COPD and spirometric evidence of airflow obstruction using either GOLD or GLI criteria is poor when assessed by the McNemar test (see online supplementary table S6).

**Table 4:** Results of spirometry in the subgroup with physician-diagnosed COPD (n=123)

	Men (n=52)	Women (n=71)	All (n=123)	p Value*
Actual median (IQR)				
FEV_1_	1.4 (1.1–1.8)	1.0 (0.7–1.1)	1.1 (0.8–1.4)	<0.001†
FVC	2.4 (2.0–3.1)	1.6 (1.3–1.9)	1.9 (1.5–2.3)	<0.001†
FEV_1_/FVC	0.6 (0.5–0.7)	0.6 (0.5–0.7)	0.6 (0.5–0.7)	0.591†
PEF	382.5 (243–519)	218 (144–290)	259 (191–380)	<0.001†
%predicted median (IQR)				
FEV_1_	63.5 (50.9–73.4)	64.2 (51.7–79.9)	64.2 (51.3–76.4)	0.609†
FVC	77.4 (64.2–94.1)	87.6 (70.4–101.0)	82.8 (68.2–99.8)	0.040†
Spirometry %(N)				
Normal	7.7 (4)	8.5 (6)	8.1 (10)	0.959‡
Restrictive	15.4 (8)	16.9 (12)	16.3 (20)	
Obstructive	76.9 (40)	74.7 (53)	75.6 (93)	
Obstructive spirometry§ %(N)				
Mild	10.0 (4)	20.8 (11)	16.1 (15)	0.190¶
Moderate	60.0 (24)	56.6 (30)	58.1 (54)	
Severe	27.5 (11)	20.8 (11)	23.7 (22)	
Very severe	2.5 (1)	1.9 (1)	2.2 (2)	
FEV_1_ %(N)				
Below LLN	48.1 (25)	33.8 (24)	39.8 (49)	0.137**
Normal range	51.9 (27)	66.2 (47)	60.2 (74)	
Above ULN	0.0 (0)	0.0 (0)	0.0 (0)	
FEV_1_ Z-score				
median (IQR)	1.6 (1.2–2.2)	1.3 (0.7–2.0)	1.5 (0.9–2.0)	0.039†
FVC %(N)				
Below LLN	30.8 (16)	14.1 (10)	21.1 (26)	0.043**
Normal range	69.2 (36)	84.5 (60)	78.1 (96)	
Above ULN	0.0 (0)	1.4 (1)	0.8 (1)	
FVC Z-score				
median (IQR)	1.1 (0.3–1.8)	0.6 (0.0–1.2)	0.8 (0.0–1.6)	0.008†
Oxygen saturation				
median (IQR)	97 (96–98)	97 (95–98)	97 (95–98)	0.521†

*Comparison of men and women.

†Mann–Whitney U test.

‡χ^2^ test.

§This is based on the 93 participant subsample with obstructive spirometry.

¶Kruskal–Wallis test.

**Fisher's exact test.

LLN, lower limit of normal; PEF, peak expiratory flow; ULN, upper limit of normal.

### Assessment of lung function in an HRG

**Figure 4:**
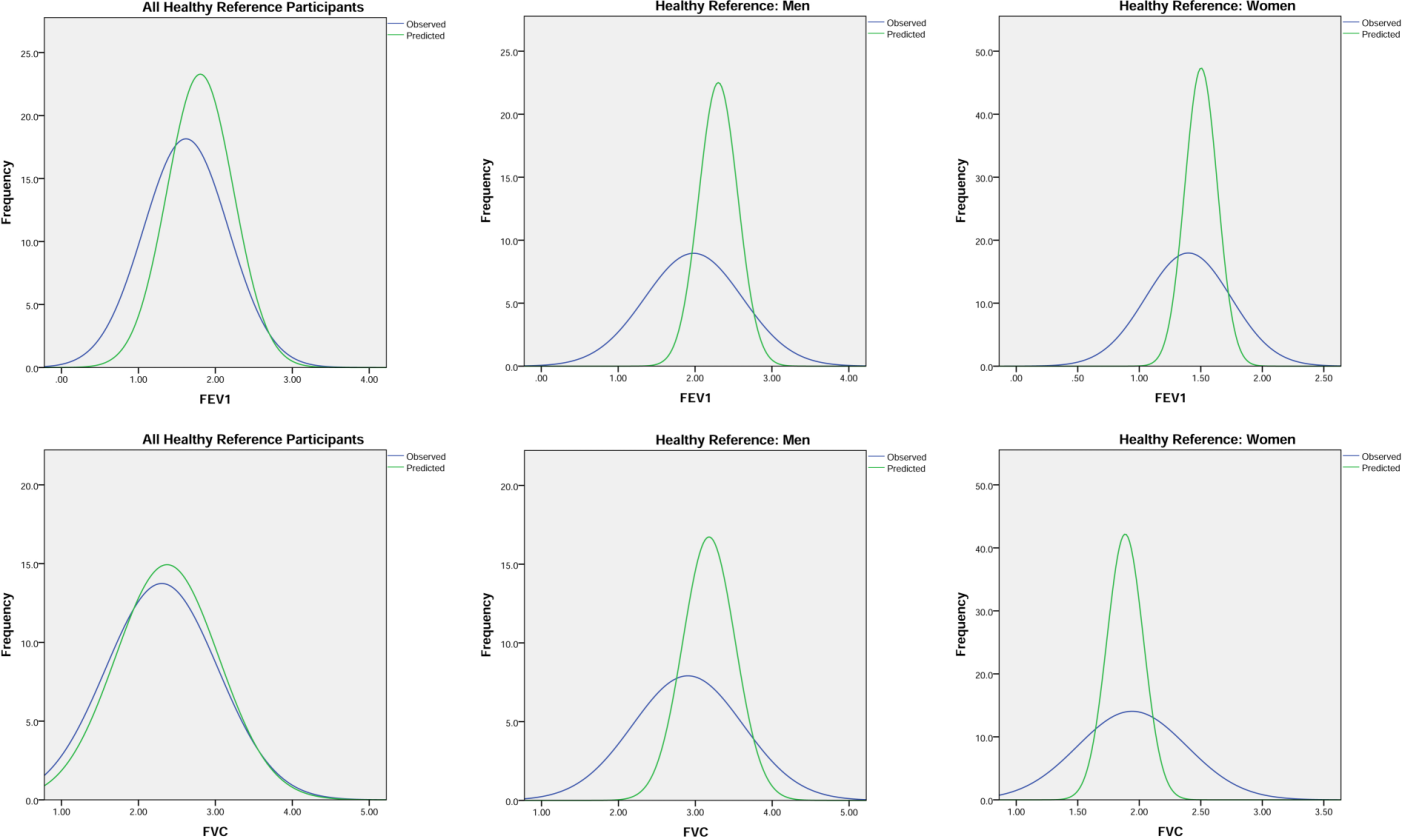
Distribution curves of FEV_1_ and FVC of participants in the healthy reference group (all, men and women) measured (blue) and predicted (green).

**Figure 5:**
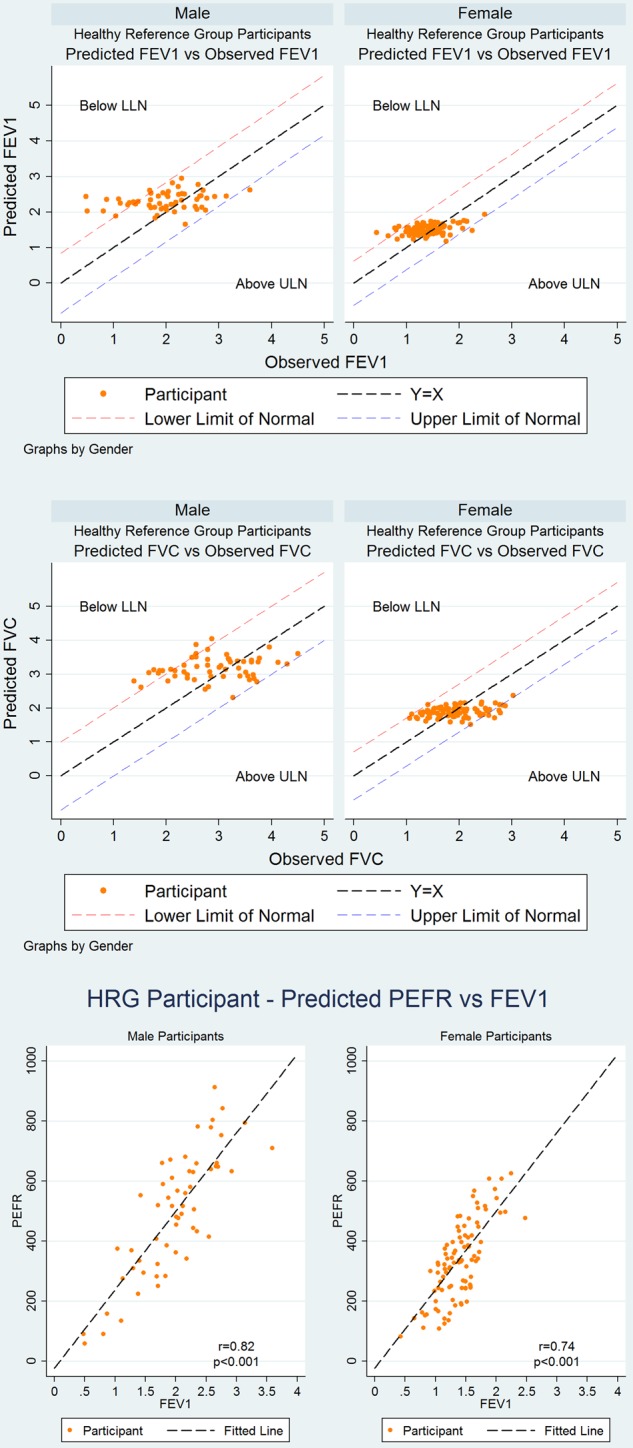
Scatter plots of spirometry and peak expiratory flow in the healthy reference group.

Figure 1 shows the derivation of the HRG which comprised 20.5% (151/737) of the spirometry cohort (table 5). The distribution of measured and predicted FEV_1_, FVC and PEF in this group, by gender, are shown in figure 4 and table 5, with scatter plots of measured versus predicted FEV_1_ and FVC by gender in figure 5.

**Table 5:** Results of spirometry in healthy reference group of participants (n=151**)**

	Men (n=57)	Women (n=94)	All (n=151)	p Value*
Actual median (IQR)				
FEV_1_	2.0 (1.7–2.4)	1.4 (1.2–1.6)	1.5 (1.2–2.0)	<0.001†
FVC	2.9 (2.4–3.5)	1.9 (1.6–2.2)	2.1 (1.8–2.8)	<0.001†
FEV_1_/FVC	0.7 (0.6–0.8)	0.7 (0.7–0.8)	0.7 (0.6–0.8)	0.244†
PEF	515 (340–647)	329.5 (243–417)	367 (263–515)	<0.001†
%predicted median (IQR)				
FEV_1_	90.1 (67.6–103.8)	93.8 (78.6–106.0)	91.6 (76.0–106.0)	0.154†
FVC	92.3 (72.0–107.7)	101.2 (85.2–121.7)	97.5 (80.6–115.2)	0.006†
Spirometry %(N)				
Normal	38.6 (22)	44.7 (42)	42.4 (64)	0.764‡
Restrictive	14.0 (98)	12.8 (12)	13.3 (20)	
Obstructive	47.4 (27)	42.6 (40)	44.4 (67)	
Obstructive spirometry§ %(N)				
Mild	48.2 (13)	62.5 (25)	56.7 (38)	0.137¶
Moderate	33.3 (9)	32.5 (13)	32.8 (22)	
Severe	11.1 (3)	5.0 (2)	7.5 (5)	
Very severe	7.4 (2)	0.0 (0)	3.0 (2)	
FEV_1_ %(N)				
Below LLN	21.1 (12)	5.3 (5)	11.3 (17)	0.008**
Normal range	77.2 (44)	93.6 (88)	87.4 (132)	
Above ULN	1.8 (1)	1.1 (1)	1.3 (2)	
FEV_1_ Z-score				
median (IQR)	0.5 (−0.2–1.6)	0.3 (−0.2–0.9)	0.3 (−0.2–1.0)	0.071†
FVC %(N)				
Below LLN	19.3 (11)	1.1 (1)	8.0 (12)	<0.001**
Normal range	79.0 (45)	91.5 (86)	86.8 (131)	
Above ULN	1.8 (1)	7.5 (7)	5.3 (8)	
FVC Z-score				
median (IQR)	0.4 (−0.4–1.5)	−0.1 (−0.9–0.6)	0.1 (−0.7–0.9)	0.004†
Oxygen saturation				
median (IQR)	98 (96–98)	98 (97–98)	98 (96–98)	0.970†

*Comparison of men and women.

†Mann–Whitney U test.

‡χ^2^ test.

§This is based on the 67 participant subsample with obstructive spirometry.

¶Kruskal–Wallis test.

**Fisher's exact test.

LLN, lower limit of normal; PEF, peak expiratory flow; ULN, upper limit of normal.

Approximately half of the HRG (men: 47.4%, 27/57; women: 42.6%, 40/94) had a spirometry definition of airflow obstruction by GOLD criteria (table 5) yet did not fulfil the requirements for a diagnosis of COPD through lack of symptoms. Interestingly 19.2% (29/151) fulfilled a spirometry definition of at least moderate COPD using NICE criteria (obstructive spirometry and an FEV_1_ <80% predicted). The measured best PEF median (IQR) for this group was 367 (263–515) L/min, significantly higher in men (515 (340–647) L/min) than in women (329.5 (243–417) L/min) (p<0.001), and highly correlated with FEV_1_ (figure 5). When applying the GLI criteria to HRG only 17.5% (10/57) men and 16% (15/94) women (see online supplementary table 5) fulfilled criteria for airflow obstruction suggesting that GLI offered superiority to GOLD in spirometry interpretation in this age group.

The measured spirometry values in HRG were compared with equation-derived^15^ predicted values based on gender and height using three different accepted approaches: percentage predicted value, LLN and Z scores (table 5). The median (IQR) percentage predicted value FEV_1_ in HRG was 90.1% (67.6–103.8%) in men and 93.8% (78.6–106.0%) in women. The measured FEV_1_ fell below LLN in 11.3% (17/151) of participants with a large gender difference (men: 21.1%, 12/57; women: 5.3%, 5/94; p=0.008). A significant gender difference was also found for the proportion of measured FVC falling below LLN with observed gender difference in the median Z-scores (table 5).

## Discussion

This study presents the first evaluation of respiratory symptomatology, respiratory disease prevalence and objectively measured lung function in a large UK population-based single-year birth cohort of 85 year olds. It provides insight into the burden of respiratory disease and degree of respiratory impairment in very old people in an urban setting, and illustrates a population with substantial environmental exposures and smoking history, even in women. Furthermore, despite the higher rate of cognitive impairment with age, 93% of our cohort performed spirometry and of these 98% did so successfully which challenges reluctance to use spirometry in the very old and dispels misconceptions that they cannot perform spirometry successfully.

The participants are long-lived, and survivors of some of the most remarkable historical periods of our time, starting in the year of their birth immediately post World War I and the 1918 Spanish influenza pandemic. There were high levels of deprivation, and unemployment across Britain reached 17% in 1921. This period was pre welfare state, Housing Act (1930), Clean Air Act and widespread use of penicillin (1940). Many of the participants would have been nearing retirement age when the 1986 WHO: Ottawa Charter for Health Promotion was introduced—smoking rates are particularly high for men.

It is therefore not unexpected that a high prevalence of physician-diagnosed COPD (16.7%) was identified compared with previous self-reports of COPD of 10% in 65–74 year olds in the 2010 Health Survey for England.^17^ Nevertheless there were signs of potential misdiagnosis of COPD with a significant proportion of those with physician-diagnosed COPD having no evidence of airflow obstruction on spirometry, no smoking or occupational history and minimal symptoms. At the same time, a high proportion of our HRG fulfilled spirometry criteria for COPD using current GOLD/NICE guidelines, though use of LLN and GLI criteria rather than GOLD or NICE guidelines might reduce levels of misdiagnosis.

The risk of respiratory impairment increases with age due to the cumulative lifetime effect of environmental insults from active and passive cigarette smoking, air pollution, occupational dusts and infections.^18^ ^19^ When this risk is added to the changes which occur in the respiratory system as part of normal ageing, including reduced ventilatory control, reduced respiratory muscle strength, increased compliance and less favourable respiratory mechanics due to reduced movement of the chest wall,^7^ it is not surprising that symptoms of cough, wheeze and dyspnoea are common in older people. All of these factors are likely to reduce measured lung function, which has been shown to be an independent risk factor for frailty and death.^20–22^ Distinguishing physiological age-related loss of lung function from a pathological disease process in the lungs is further complicated by a reduced perception of respiratory symptoms that occurs with increasing age as demonstrated by significantly reduced awareness of measured bronchospasm after a methacholine challenge in older compared with younger patients.^23^ Despite the high prevalence of chronic lung disease and respiratory symptoms, we found a significant proportion, 50% of men and 40% of women, with no reported limitations due to breathlessness suggesting many are either able to function very well or have a poor perception of symptoms.

The strengths of this work are the comprehensive assessment of respiratory health and lung disease in a large population-based cohort of 85 year olds, including those in institutional care and those with cognitive impairment, in a stable urban setting and with little ethnic diversity. The cohort of >800 participants was achieved through engagement with 83% of the general practices in the area and a consent rate of almost 60% in those approached to participate. Previous studies of respiratory health in older subjects have relied on self-reported diagnoses whereas in our study the use of general practice records significantly improves the validity of our findings.^24^ ^25^ Furthermore by conducting spirometry in the participant's place of residence using trained research nurses we were able to achieve a very high uptake of this assessment, in contrast to the known selection bias if participants had been required to attend a clinic for assessment. Although participants opting in for the health assessment were not a random sample of those eligible, there was little evidence to suggest they had more or less respiratory disease than those refusing the health assessment. In addition they were sociodemographically representative of their England and Wales birth cohort.^4^ A potential limitation of the study is that those who agreed to participate may be healthier and less frail than those who declined to participate and those with cognitive impairment may have been under-represented. Although some information was collected about why those invited declined to participate, we obviously do not have objective data on their respiratory health or disease burden. However the prevalence of COPD of 16.7% in those who agreed to MDHA and GPRR (n=845) was very similar to the prevalence of 16.5% reported previously in all participants with GPRR data (n=1030),^4^ suggesting that in terms of COPD, those agreeing to MDHA had similar respiratory health profiles to the larger study population. While 85 year olds in this urban area in North-East England are sociodemographically and ethnically similar to the same birth cohort in England and Wales as a whole, they may differ from those in other parts of the world.

This study has revealed a substantial burden of respiratory symptoms and respiratory disease, particularly COPD, in a cohort of the very old aged 85 years; a group with substantial environmental exposures recorded through smoking and occupational exposure, which are known risk factors for lung disease. Despite these observations, we show a good proportion of participants functioning well with no respiratory symptoms or diagnoses. Lung function tests revealed only 75.6% of the COPD group satisfied spirometry criteria whereas 44% of the healthy group satisfied spirometry criteria for COPD using GOLD criteria. Healthcare professionals need to recognise that spirometry can be reliably assessed in the vast majority of this age group but care is needed as to how this is interpreted. Current definitions of COPD based on spirometry may lead to overdiagnosis in a group with transient symptoms and ‘normal’ lung ageing, whereas at the same time failure to use spirometry to assess symptoms in this age group may lead to mislabelling those with breathlessness or cough as having COPD when there are other explanations.

## Supplementary Material

Web supplement
