# Supplementary material for: Respiratory health and disease in a UK population-based cohort of 85 year olds: The Newcastle 85+ Study
Source: Thorax. 2016 Jan 5;71(3):255–66. doi: 10.1136/thoraxjnl-2015-207249 (PMC4789822; doi:10.1136/thoraxjnl-2015-207249)
Supplement: Web supplement [file thoraxjnl-2015-207249-s1.pdf]

Appendices

Supplementary Methods

Participant Recruitment

The only exclusions from the initial invitation to participate in the Newcastle 85+ Study were people deemed by their general practitioner to have end-stage terminal illness or whose behaviour might prove a threat to a research nurse visiting alone. The multidimensional health assessment (MDHA) was performed by a trained research nurse in their usual place of residence over three visits conducted over the course of one month with the respiratory assessments performed on the second or third visit. In addition, the research nurses reviewed participants' medical records in a general practice record review (GPRR) to obtain information on current and past diagnoses and current medication. Both computerised and paper records were examined, including hospital correspondence. In the UK, patients are registered with a single general practice that acts as a gatekeeper to secondary care and receives details of all hospital admissions and outpatient attendances.

Respiratory diseases and medications identified from the GPRR

The predetermined checklist of chronic respiratory diseases included COPD; Asthma; Bronchiectasis; Pulmonary Fibrosis/Fibrosing Alveolitis; Asbestosis; Pneumoconiosis and Tuberculosis. The list of respiratory medications included inhaled short or long acting beta-2 adrenoreceptor agonists, inhaled short or long acting muscarinic antagonists, inhaled corticosteroids either as single agent or as part of a combination with long acting beta-2 adrenoreceptor agonists, oral corticosteroids, oral leukotriene receptor antagonists, oral theophylline and supplemental oxygen.

Chronic diseases comprising the comorbidity measure

The original disease count comprised 18 diseases: COPD, other respiratory disease, hypertension, ischaemic heart disease, cerebrovascular disease, peripheral vascular disease, heart failure, atrial fibrillation, arthritis, osteoporosis, diabetes mellitus, thyroid disease, cancer excluding non-melanoma skin cancer, eye disease, dementia, Parkinson's Disease, anaemia and renal impairment. For the non-respiratory disease count we excluded COPD and other respiratory disease (maximum count = 16).

Research in context

Evidence before this study

We undertook a review of literature on respiratory epidemiology in older ages in Scopus and PubMed over the previous 15 years using the search terms "respiratory", "epidemiology", "old age", and "lung function". We then focussed on studies that were solely in people aged 75 years and over or covered a wider age range but with a reasonable number aged 75 years and over. We found only 2 studies reporting respiratory function in people aged 85 years or over: the Swedish Twin Study with 808 participants, 26 of which were aged 86 and over; [1] and the Danish 1905 birth cohort with 592 participants aged 93 at assessment. [2]

Added value of this study

Our prevalence of physician-diagnosed COPD (16.6%) is higher than previous self-reports of COPD in 65-74 year olds (men: 11%, women: 10%) in the 2010 Health Survey for England (HSE).[3] Interestingly in the 2010 HSE, the prevalence of COPD was less in those aged over 75 (men: 9%, women: 4%), possibly because it excludes people in institutions. Our findings confirm that the very old population is living with a very high prevalence of chronic lung disease particularly COPD. It is unclear if this represents environmental exposures and is limited to an urban setting in the North East or is a national situation in the United Kingdom, though 23% of the study population were born outside of the North East.[4]

The current way that lung function is categorised as normal or abnormal in clinical practice is generally based on the definitions presented in the GOLD criteria.[5] If the measured FEV_1/FVC ratio is less than 0.7 then a diagnosis of airflow obstruction is made by GOLD criteria and if additionally the FEV_1 is less than 80% predicted then a symptomatic individual fulfils the definition for moderate COPD in the UK NICE guidelines.[6] The measured values are reported as a percentage of predicted values based on equation derived reference values for FEV_1 and FVC for age, height and gender. There is however potential risk in applying this approach to very old people for two main reasons. Firstly the reference values in this age group are derived from an extrapolation of measurements taken in healthy people of a younger age and therefore may not reflect the physiological changes that occur to lung function with very advanced ageing. Secondly, this risks misdiagnosis of normal ageing as pathological lung disease and might lead to inappropriate diagnostic labelling and use of medications that can have significant side effects.

The accuracy of lung function impairment by GOLD criteria and Lower Limit of Normal (LLN) in diagnosing COPD has been compared against the gold standard of an expert clinical panel assessment in a cohort of 405 younger-old (median age= 72, IQR=69-77) with findings that the GOLD criteria were found to over diagnose COPD and the LLN approach to under diagnose COPD.[7] However this cohort did not contain participants as old as 85 years, hence our results add significantly to the evidence for over-diagnosis by GOLD criteria. Additionally we found that the LLN approach would reduce, though not eliminate, over-diagnosis. In the Danish 1905 birth cohort, predictive equations derived from a general US population [8] were superior at predicting survival compared to those generated specifically from an elderly population,[2] although others have reported that spirometry reference ranges derived from a specific cohort of 458 healthy never smokers aged over 65 were superior to those derived by extrapolation of reference ranges from the general population.[9] Nevertheless the latter two studies did not assess accuracy of their spirometry definitions in specific subgroups of their study population namely those with a diagnosis of COPD or a healthy reference group, as has been done in our study. Previous findings in healthy participants aged over 70 years demonstrated that 35% had obstructive spirometry by GOLD definition and that in their small subgroup (n=28) >80 years of age, one third had both a FEV_1/FVC ratio <0.7 and FEV_1 percentage predicted <80%. [10] Our findings strengthen this evidence by including a much larger and significantly older cohort.

Implications of all the available evidence

Our findings reveal substantial discordance between a physician diagnosis of COPD and confirmatory spirometry evidence in the very old that may have important implications for practice. By both GOLD definitions and the UK based NICE definitions, we found that only 75.6% of the COPD population satisfied spirometry criteria for a COPD diagnosis. Firstly this raises the possibility that spirometry may not have been

used to establish a COPD diagnosis in the population due to a perception that they cannot perform it adequately. Our study has however demonstrated that adequate reproducible blows conforming to ATS/ERS standards could be produced by over 90% of the very old and under the supervision of a trained research nurse rather than a clinical physiologist. Secondly, only 20.3% of the COPD cohort, were being treated with a long acting muscarinic antagonist (LAMA), which is usually considered the first line regular bronchodilator agent in COPD management. This suggests that 85 year olds with a COPD diagnosis may not be receiving standard therapies for their condition. Finally that just under half of our healthy reference group fulfilled criteria for airflow obstruction using GOLD and NICE definitions suggests that respiratory symptoms such as cough or breathlessness, which may be due to transient respiratory infection or non-respiratory causes, might be misdiagnosed as COPD by GOLD/NICE spirometry criteria and therefore given unnecessary treatment. The lower limit of normal (LLN) approach appears to reduce the risk of false positives in an otherwise healthy population but further research with longitudinal measures of lung function and diagnoses is necessary to validate this.

Supplementary Table 1: Comparison of the groups included and excluded in the spirometry cohort.**

		Excluded from Spirometry Cohort (n=108)	Included in Spirometry cohort (n=737)	p-value* -
Sex	Female	75.9 (82)	60.2 (444)	0.002[2]
Ethnicity	White	98.1 (104)	99.9 (735)	0.043[2]
Living arrangements % (N)	Standard housing	46.3 (50)	81.6 (601)	<0.001[2]
	Sheltered housing	13.0 (14)	12.8 (94)	
	Institutional care	40.7 (44)	5.7 (42)	
Smoking % (N)	Never	41.9 (44)	35.0 (257)	0.375[2]
	Former	53.3 (56)	59.2 (435)	
	Current	4.8 (5)	5.9 (43)	
Occupational Exposures % (N)	Heavy Industry	18.1 (13)	26.7 (196)	0.111[2]
	Coal mining	2.7 (2)	4.5 (33)	0.763[3]
	Chemical industry	12.7 (9)	6.1 (45)	0.035[2]
	Asbestos exposure	5.7 (4)	12.6 (92)	0.121[3]
Respiratory symptoms % (N)	Cough	26.9 (21)	26.7 (196)	0.961[2]
	Wheeze	20.8 (16)	22.2 (163)	0.783[2]
	Sputum production	26.0 (20)	33.6 (247)	0.177[2]
MRC Dyspnoea Score % (N)	1	40.5 (15)	44.8 (251)	0.257[1]
	2	13.5 (5)	16.0 (90)	
	3	13.5 (5)	19.1 (107)	
	4	21.6 (8)	15.9 (89)	
	5	10.8 (4)	4.3 (24)	
Respiratory diagnoses % (N)	COPD	15.7 (17)	16.7 (123)	0.804[2]
	Asthma	7.4 (8)	4.1 (30)	0.118[2]
	Bronchiectasis	0.9 (1)	2.0 (15)	0.708[3]
	Pulmonary Fibrosis	0.9 (1)	0.0 (0)	0.128[3]
	Asbestosis	0.0 (0)	0.7 (5)	1.000[3]
	Pneumoconiosis	0.0 (0)	0.5 (4)	1.000[3]
	Tuberculosis	2.8 (3)	5.0 (37)	0.465[3]
Medications	Inhaled short acting β-2 adrenoreceptor agonists	11.1 (12)	10.5 (77)	0.834[2]
	Inhaled muscarinic antagonists	3.7 (4)	3.8 (28)	1.000[3]
	Oral Theophylline	0.0 (0)	0.5 (4)	1.000[3]
	Combination short acting bronchodilators	0.0 (0)	0.3 (2)	1.000[3]
	Inhaled Corticosteroids	4.6 (5)	7.2 (53)	0.325[2]
	Combination inhaled Corticosteroids and long acting β-2 adrenoreceptor agonists	0.9 (1)	2.2 (16)	0.712[3]
	Oral leukotriene receptor antagonists	0.0 (0)	0.3 (2)	1.000[3]
	Oral mucolytics	0.0 (0)	0.4 (3)	1.000[3]
At least 1 Respiratory Medication - % (N)		12.0 (13)	13.8 (102)	0.610[2]
Disease count - Median (IQR)		5 (4 - 6)	4 (3 - 6)	0.178[1]

* Comparison of Men and Women; ** Denominators may vary due to missing values; [1] Mann-Whitney test; [2] Chi-square test; [3] Fisher-exact test

Supplementary Table 2: Spirometry Definitions

Spirometry Definition		
Normal	$FEV_1/FVC > 0.7$	FEV1 ≥ 80% predicted
Restrictive	$FEV_1/FVC > 0.7$	FEV1 ≤ 80% predicted
Obstructive	$FEV_1/FVC < 0.7$	
Obstructive Spirometry Grading Definition		
Mild	$FEV_1/FVC < 0.7$	FEV1 ≥ 80% predicted
Moderate	$FEV_1/FVC < 0.7$	50% ≤ FEV1 < 80% predicted
Severe	$FEV_1/FVC < 0.7$	30% ≤ FEV1 < 50% predicted
Very Severe	$FEV_1/FVC < 0.7$	FEV1 < 30% predicted
Limits of Normal Range	FEV_1	FVC
Men	FEV_1Pred +/- (0.51*1.645)	FVCPred +/- (0.61*1.645)
Women	FEV_1Pred +/- (0.38*1.645)	FVCPred +/- (0.43*1.645)
Use + for Upper Limit of Normal (ULN) and - for Lower Limit of Normal (LLN)		
Z-Score	FEV_1	FVC
Men	(FEV_1Pred - FEV1 Actual)/0.51	(FVCPred - FVCActual)/0.61
Women	(FEV_1Pred - FEV1 Actual)/0.38	(FVCPred - FVCActual)/0.43
Range	Upper Limit of Normal > 1.645	Lower Limit of Normal < -1.645

Supplementary Table 3: Results of Spirometry in the cohort completing spirometry with adequate reproducible blows and demi-span available for calculation of predicted blows (n=737) based on Global Lungs Initiative (GLI) prediction models

		Men<br>(n=293)	Women<br>(n=444)	All<br>(n=737)	p-value*
Actual<br>Spirometry<br>Median (IQR)	FEV1<br>FVC<br>FEV1/FVC<br>PEFR	1.8 (1.4 - 2.2)<br>2.7 (2.2 - 3.2)<br>0.7 (0.6 - 0.8)<br>441 (323 - 604)	1.2 (1.0 - 1.5)<br>1.8 (1.4 - 2.1)<br>0.7 (0.6 - 0.8)<br>283 (196 - 362)	1.4 (1.1 - 1.8)<br>2.0 (1.6 - 2.6)<br>0.7 (0.6 - 0.8)<br>328 (233 - 450)	<0.001[1]<br><0.001[1]<br>0.006[1]<br><0.001[1]
%predicted<br>Median (IQR)	FEV1<br>FVC	74.3 (58.7 - 88.6)<br>80.8 (67.2 - 95.9)	72.4 (59.4 - 87.2)<br>80.4 (64.9 - 94.0)	73.2 (58.9 - 87.7)<br>80.6 (66.1 - 94.7)	0.457[1]<br>0.162[1]
Spirometry<br>% (N)	Normal<br>Restrictive<br>Obstructive	52.9 (155)<br>22.2 (65)<br>24.9 (73)	52.5 (233)<br>24.1 (107)<br>23.4 (104)	52.7 (388)<br>23.3 (172)<br>24.0 (177)	0.800[2]
FEV1<br>%(N)	Below LLN<br>Normal range<br>Above ULN	39.9 (117)<br>59.7 (175)<br>0.3 (1)	42.1 (187)<br>57.4 (255)<br>0.5 (2)	41.3 (304)<br>58.3 (430)<br>0.4 (3)	0.823[3]
FEV1 Z-Score	Median (IQR)	-1.3 (-2.1 - -0.6)	-1.4 (-2.1 - -0.7)	-1.4 (-2.1 - -0.6)	0.248[1]
FVC<br>%(N)	Below LLN<br>Normal range<br>Above ULN	31.4 (92)<br>67.6 (198)<br>1.0 (3)	31.5 (140)<br>68.2 (303)<br>0.2 (1)	31.5 (232)<br>68.0 (501)<br>0.5 (4)	0.442[3]
FVC Z-Score	Median (IQR)	-1.1 (-1.9 - -0.2)	-1.0 (-1.8 - -0.3)	-1.1 (-1.8 - -0.3)	0.727[1]
Oxygen Saturation	Median (IQR)	97 (96 - 98)	97 (96 - 98)	97 (96 - 98)	0.513[1]

* Comparison of Men and Women; [1] Mann-Whitney test; [2] Chi-square test; [3] Fisher-exact test;

Supplementary Table 4: Results of Spirometry in the sub-group with Physician diagnosed of COPD (n=123) based on Global Lungs Initiative (GLI) prediction models

		Men (n=52)	Women (n=71)	All (n=123)	p-value* -
Actual Median (IQR)	FEV1	1.4 (1.1 - 1.8)	1.0 (0.7 - 1.1)	1.1 (0.8 - 1.4)	<0.001[1]
	FVC	2.4 (2.0 - 3.1)	1.6 (1.3 - 1.9)	1.9 (1.5 - 2.3)	<0.001[1]
	FEV1/FVC	0.6 (0.5 - 0.7)	0.6 (0.5 - 0.7)	0.6 (0.5 - 0.7)	0.591[1]
	PEFR	382.5 (243 - 519)	218 (144 - 290)	259 (191 - 380)	<0.001[1]
%predicted Median (IQR)	FEV1	60.7 (48.8 - 69.1)	56.5 (43.4 - 68.9)	58.6 (44.8 - 69.0)	0.313[1]
	FVC	73.8 (62.0 - 91.1)	71.8 (59.5 - 85.0)	73.2 (60.3 - 86.8)	0.221[1]
Spirometry %(N)	Normal	26.9 (14)	23.9 (17)	25.2 (31)	0.928[2]
	Restrictive	25.0 (13)	25.4 (18)	25.2 (31)	
	Obstructive	48.1 (25)	50.7 (36)	49.6 (61)	
FEV1 %(N)	Below LLN	69.2 (36)	74.7 (53)	72.4 (89)	0.545[3]
	Normal range	30.8 (16)	25.4 (18)	27.6 (34)	
	Above ULN	0.0 (0)	0.0 (0)	0.0 (0)	
FEV1 Z-Score	Median (IQR)	-2.0 (-2.5 - -1.6)	-2.3 (-2.9 - -1.6)	-2.1 (-2.8 - -1.6)	0.128[1]
FVC %(N)	Below LLN	46.2 (24)	45.1 (32)	45.5 (56)	1.000[3]
	Normal range	53.9 (28)	54.9 (39)	54.5 (67)	
	Above ULN	0.0 (0)	0.0 (0)	0.0 (0)	
FVC Z-Score	Median (IQR)	-1.5 (-2.2 - -0.5)	-1.5 (-2.1 - -0.8)	-1.5 (-2.2 - -0.7)	0.560[1]
Oxygen Saturation	Median (IQR)	97 (96 - 98)	97 (95 - 98)	97 (95 - 98)	0.521[1]

* Comparison of Men and Women; [1] Mann-Whitney test; [2] Chi-square test; [3] Fisher-exact test;

Supplementary Table 5: Results of Spirometry in healthy reference group of participants (n=151) based on Global Lungs Initiative (GLI) prediction values

		Men (n=57)	Women (n=94)	All (n=151)	p-value*
Actual Median (IQR)	FEV1	2.0 (1.7 - 2.4)	1.4 (1.2 - 1.6)	1.5 (1.2 - 2.0)	<0.001[1]
	FVC	2.9 (2.4 - 3.5)	1.9 (1.6 - 2.2)	2.1 (1.8 - 2.8)	<0.001[1]
	FEV1/FVC	0.7 (0.6 - 0.8)	0.7 (0.7 - 0.8)	0.7 (0.6 - 0.8)	0.244[1]
	PEFR	515 (340 - 647)	329.5 (243 - 417)	367 (263 - 515)	<0.001[1]
%predicted Median (IQR)	FEV1	83.9 (65.7 - 97.5)	84.0 (69.7 - 93.3)	83.9 (69.0 - 94.2)	0.997[1]
	FVC	90.3 (70.5 - 104.2)	85.0 (72.4 - 99.3)	86.4 (70.9 - 102.8)	0.602[1]
Spirometry %(N)	Normal	63.2 (36)	67.0 (63)	65.6 (99)	0.855[2]
	Restrictive	19.3 (11)	17.0 (16)	17.9 (27)	
	Obstructive	17.5 (10)	16.0 (15)	16.6 (25)	
FEV1 %(N)	Below LLN	26.3 (15)	23.4 (22)	24.5 (37)	0.857[3]
	Normal range	71.9 (41)	75.5 (71)	74.2 (112)	
	Above ULN	1.8 (1)	1.1 (1)	1.3 (2)	
FEV1 Z-Score	Median (IQR)	-0.9 (-1.7 -0.1)	-0.9 (-1.6 - -0.4)	-0.9 (-1.6 - -0.3)	0.918[1]
FVC %(N)	Below LLN	26.3 (15)	21.3 (20)	23.2 (35)	0.302[3]
	Normal range	73.7 (42)	78.7 (74)	76.8 (116)	
	Above ULN	0.0 (0)	0.0 (0)	0.0 (0)	
FVC Z-Score	Median (IQR)	-0.5 (-1.7 - 0.2)	-0.8 (-1.4 - 0.0)	-0.7 (-1.6 - 0.1)	0.845[1]
Oxygen Saturation	Median (IQR)	98 (96 - 98)	98 (97 - 98)	98 (96 - 98)	0.970[1]

* Comparison of Men and Women; [1] Mann-Whitney test; [2] Chi-square test; [3] Fisher-exact test;

Supplementary Table 6: Level of agreement between the three methods of classification of obstructive lung function

		GP Diagnosed COPD	
		No	Yes
GOLD Obstructive	No	91.2 (312)	8.8 (30)
	Yes	76.5 (302)	23.5 (93)
	Total	83.3 (614)	16.7 (123)
			p<0.001*

		GP Diagnosed COPD	
		No	Yes
GLI Obstructive	No	88.9 (498)	11.1 (62)
	Yes	65.5 (116)	34.5 (61)
	Total	83.3 (614)	16.7 (123)
			p<0.001*

		GOLD COPD	
		No	Yes
GLI Obstructive	No	61.1 (342)	38.9 (218)
	Yes	0.0 (0)	100.0 (177)
	Total	46.4 (342)	53.6 (395)
			p<0.001*

* McNemar test

References

1. Finkel D, Reynolds CA, Emery CF*, et al.* Genetic and environmental variation in lung function drives subsequent variation in aging of fluid intelligence. *Behav Genet* 2013;43:274-85.
2. Miller MR, Thinggaard M, Christensen K*, et al.* Best lung function equations for the very elderly selected by survival analysis. *Eur Respir J* 2014;43:1338-46.
3. National Health Service N. Health Survey for England 2010, Respiratory Health. The NHS Information Centre 2011.
4. Collerton J, Davies K, Jagger C*, et al.* Health and disease in 85 year olds: baseline findings from the Newcastle 85+ cohort study. *BMJ* 2009;339:b4904.
5. GOLD. Spirometry for Health Care Providers. In: Global Initiative for Chronic Obstructive Lung Disease, ed. 2010.
6. National Institute for Health and Clinical Excellence. Chronic obstructive pulmonary disease: Management of chronic obstructive pulmonary disease in adults in primary and secondary care (partial update). In: National Health Service, ed. 2010.
7. Guder G, Brenner S, Angermann CE*, et al.* "GOLD or lower limit of normal definition? A comparison with expert-based diagnosis of chronic obstructive pulmonary disease in a prospective cohort-study". *Respir Res* 2012;13:13.
8. Hankinson JL, Odencrantz JR, Fedan KB. Spirometric reference values from a sample of the general U.S. population. *Am J Respir Crit Care Med* 1999;159:179-87.
9. Garcia-Rio F, Pino JM, Dorgham A*, et al.* Spirometric reference equations for European females and males aged 65-85 yrs. *Eur Respir J* 2004;24:397-405.
10. Hardie JA, Buist AS, Vollmer WM*, et al.* Risk of over-diagnosis of COPD in asymptomatic elderly never-smokers. *Eur Respir J* 2002;20:1117-22.
